# The genome sequence of the Lulworth Skipper, Thymelicus acteon (Rottemburg, 1775)

**DOI:** 10.12688/wellcomeopenres.21627.1

**Published:** 2024-05-15

**Authors:** Konrad Lohse, Roger Vila, Alex Hayward, Wellcome Sanger Institute Tree of Life Management, Samples and Laboratory team, Wellcome Sanger Institute Scientific Operations: Sequencing Operations, Wellcome Sanger Institute Tree of Life Core Informatics team, Tree of Life Core Informatics collective, Darwin Tree of Life Consortium

**Affiliations:** 1 Institute of Ecology and Evolution, The University of Edinburgh, Edinburgh, Scotland, UK; 2 Institut de Biologia Evolutiva, CSIC - Universitat Pompeu Fabra, Barcelona, Catalonia, Spain; 3 Department of Biosciences, University of Exeter, Penryn, England, UK

**Keywords:** Thymelicus acteon, Lulworth Skipper, genome sequence, chromosomal, Lepidoptera

## Abstract

We present a genome assembly from an individual male *Thymelicus acteon* (the Lulworth Skipper; Arthropoda; Insecta; Lepidoptera; Hesperiidae). The genome sequence is 537.0 megabases in span. Most of the assembly is scaffolded into 28 chromosomal pseudomolecules, including the Z sex chromosome. The mitochondrial genome has also been assembled and is 17.08 kilobases in length. Gene annotation of this assembly on Ensembl identified 12,813 protein-coding genes.

## Species taxonomy

Eukaryota; Opisthokonta; Metazoa; Eumetazoa; Bilateria; Protostomia; Ecdysozoa; Panarthropoda; Arthropoda; Mandibulata; Pancrustacea; Hexapoda; Insecta; Dicondylia; Pterygota; Neoptera; Endopterygota; Amphiesmenoptera; Lepidoptera; Glossata; Neolepidoptera; Heteroneura; Ditrysia; Obtectomera; Hesperioidea; Hesperiidae; Hesperiinae; Hesperiini; *Thymelicus*; *Thymelicus acteon* (Rottemburg, 1775) (NCBI:txid876078).

## Background

The Lulworth Skipper ( *Thymelicus acteon*) is a habitat specialist of warm grasslands in North Africa, Southern and Central Europe, the Middle East and Western Asia. *Thymelicus acteon* reaches its Northern range limit in the South of England where it occurs in a number of localities on south-facing limestone and chalk grasslands in Dorset, centred around the village of Lulworth where it was first recorded in the UK in 1832. It is absent from Wales, Scotland and Ireland.

The Lulworth Skipper is listed as Near Threatened on the IUCN and UK Red lists ( Fox *et al.*, 2015; Van Swaay *et al.*, 2010), but in the UK it has increased in abundance since 2014, possibly as a result of climate change. The species is univoltine with adults occurring from the end of April to August depending on locality. Eggs are laid in a row in flower-sheaths of Tor-grass ( *Brachypodium pinnatum*), the principal larval host plant in the UK, although it feeds on several other grasses across its range.

The Lulworth skipper has lower overall genetic diversity compared to its congeners *T. sylvestris* and *T. lineola* ( Mackintosh *et al.*, 2019). At the local scale in Central Europe it shows no isolation by distance, but relatively high genetic structure ( Louy *et al.*, 2007). Population connectivity has been shown to be strongly influenced by land use patterns ( Engler *et al.*, 2014). In the Western Palearctic, this species shows several geographically-structured mitochondrial lineages ( Dapporto *et al.*, 2022) as well as phenotypic variation: darker forms occur in north-west Africa, Iberia, Elba, Crete, and other eastern Mediterranean islands. The taxon *christi*, previously considered a subspecies of *T. acteon*, is now generally treated as a species endemic to the Canary Islands.

The Lulworth skipper has 28 chromosomes ( de Lesse, 1970); the genome size has been estimated as 517.4 Mb ( Mackintosh *et al.*, 2019).

## Genome sequence report

**Figure 1.**
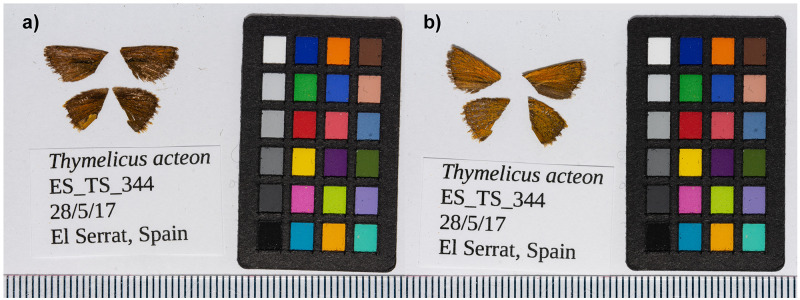
Photographs of forewings and hindwings of the *Thymelicus acteon* specimen ES_TS_344 (ilThyActe1) used for genome sequencing. **a**) Dorsal and **b**) ventral surface views of wings from the specimen.

The genome was sequenced from a male *Thymelicus acteon* ( Figure 1) collected from El Serrat, Catalunya, Spain (41.69, 2.25). A total of 42-fold coverage in Pacific Biosciences single-molecule HiFi long reads was generated. Primary assembly contigs were scaffolded with chromosome conformation Hi-C data. Manual assembly curation corrected 14 missing joins or mis-joins and removed 6 haplotypic duplications, reducing the assembly length by 0.77% and the scaffold number by 9.46%.

**Figure 2.**
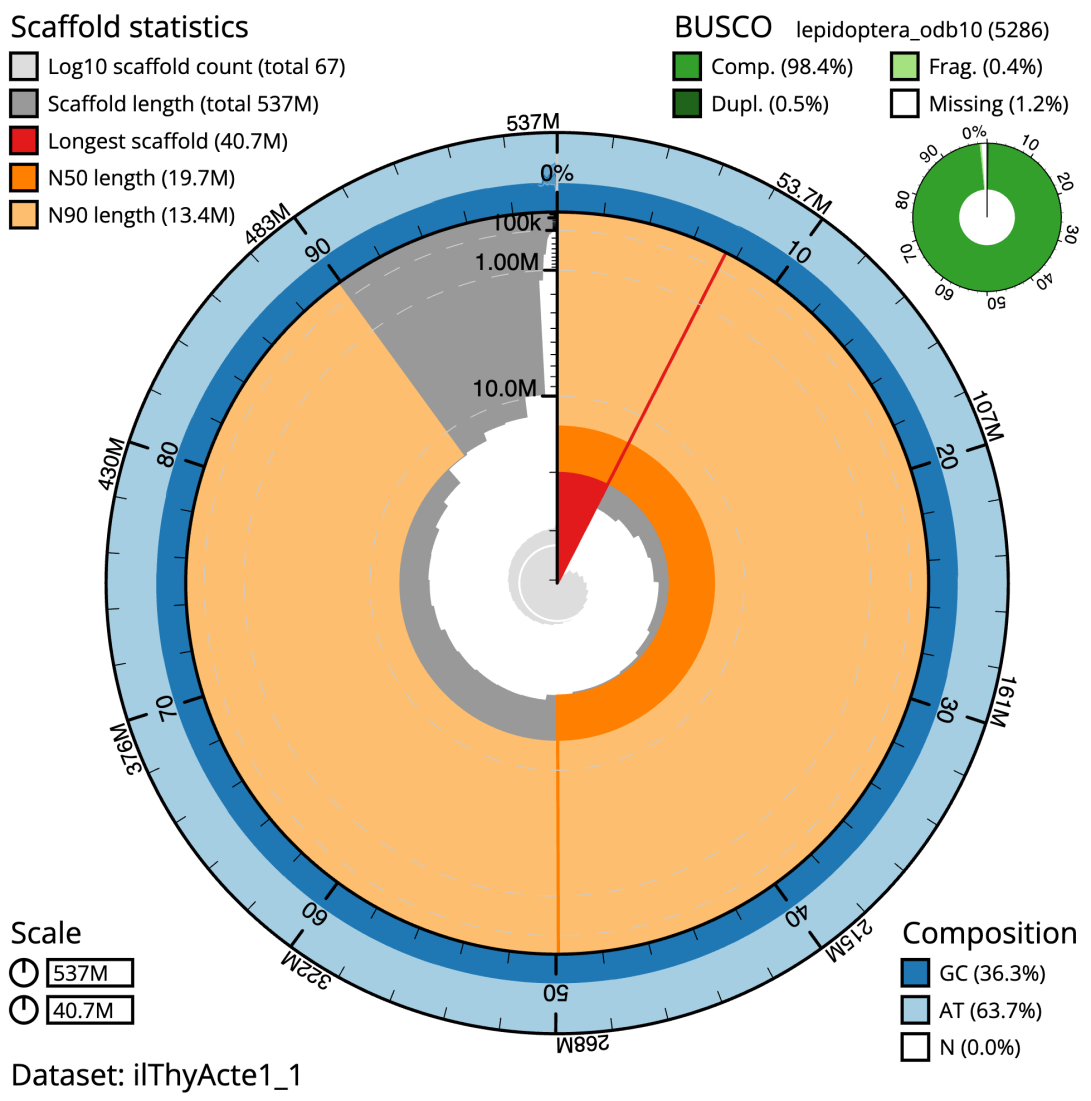
Genome assembly of *Thymelicus acteon*, ilThyActe1.1: metrics. The BlobToolKit snail plot shows N50 metrics and BUSCO gene completeness. The main plot is divided into 1,000 size-ordered bins around the circumference with each bin representing 0.1% of the 536,997,220 bp assembly. The distribution of scaffold lengths is shown in dark grey with the plot radius scaled to the longest scaffold present in the assembly (40,667,834 bp, shown in red). Orange and pale-orange arcs show the N50 and N90 scaffold lengths (19,676,428 and 13,448,605 bp), respectively. The pale grey spiral shows the cumulative scaffold count on a log scale with white scale lines showing successive orders of magnitude. The blue and pale-blue area around the outside of the plot shows the distribution of GC, AT and N percentages in the same bins as the inner plot. A summary of complete, fragmented, duplicated and missing BUSCO genes in the lepidoptera_odb10 set is shown in the top right. An interactive version of this figure is available at https://blobtoolkit.genomehubs.org/view/ilThyActe1_1/dataset/ilThyActe1_1/snail.

**Figure 3.**
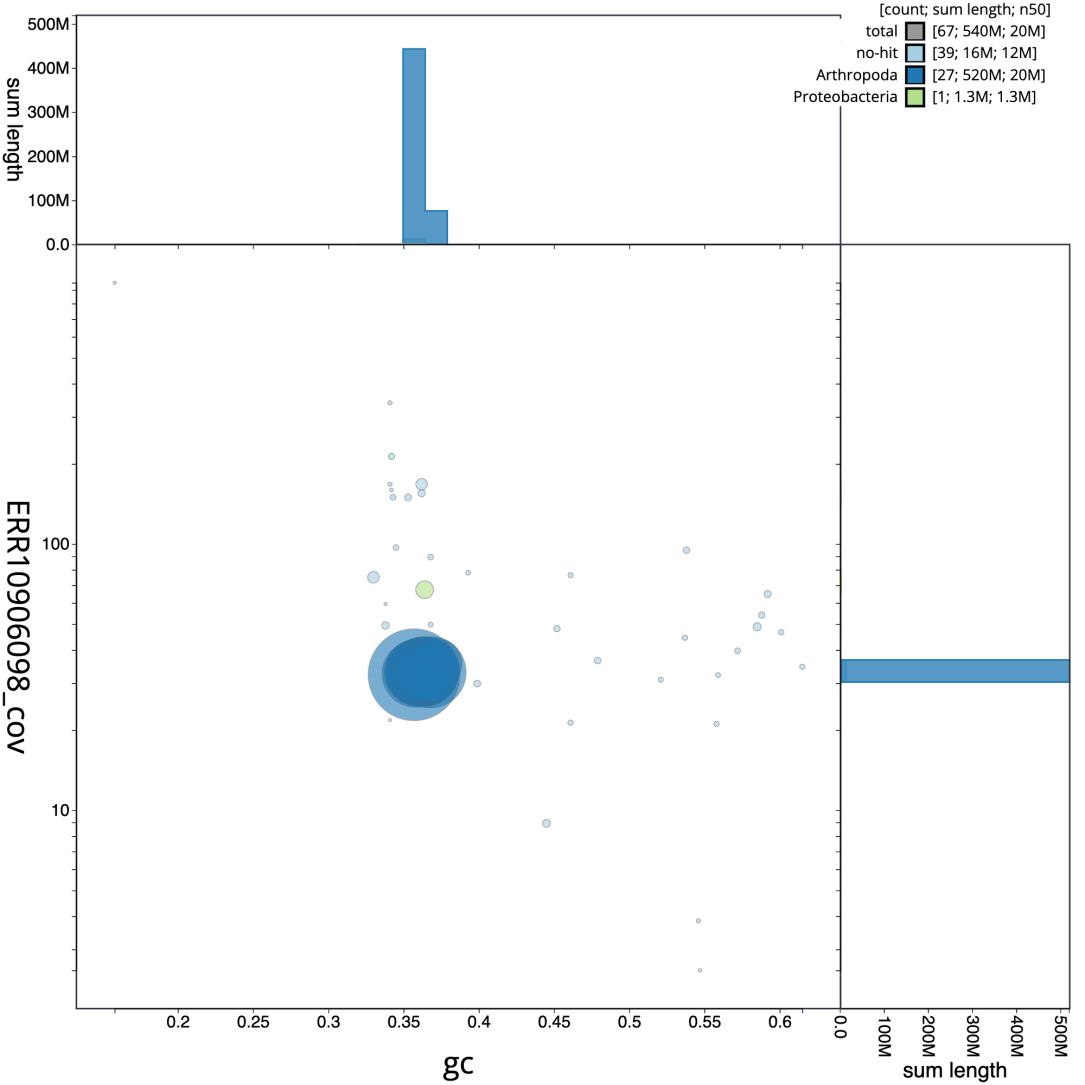
Genome assembly of *Thymelicus acteon*, ilThyActe1.1: BlobToolKit GC-coverage plot. Sequences are coloured by phylum. Circles are sized in proportion to sequence length. Histograms show the distribution of sequence length sum along each axis. An interactive version of this figure is available at https://blobtoolkit.genomehubs.org/view/ilThyActe1_1/dataset/ilThyActe1_1/blob.

**Figure 4.**
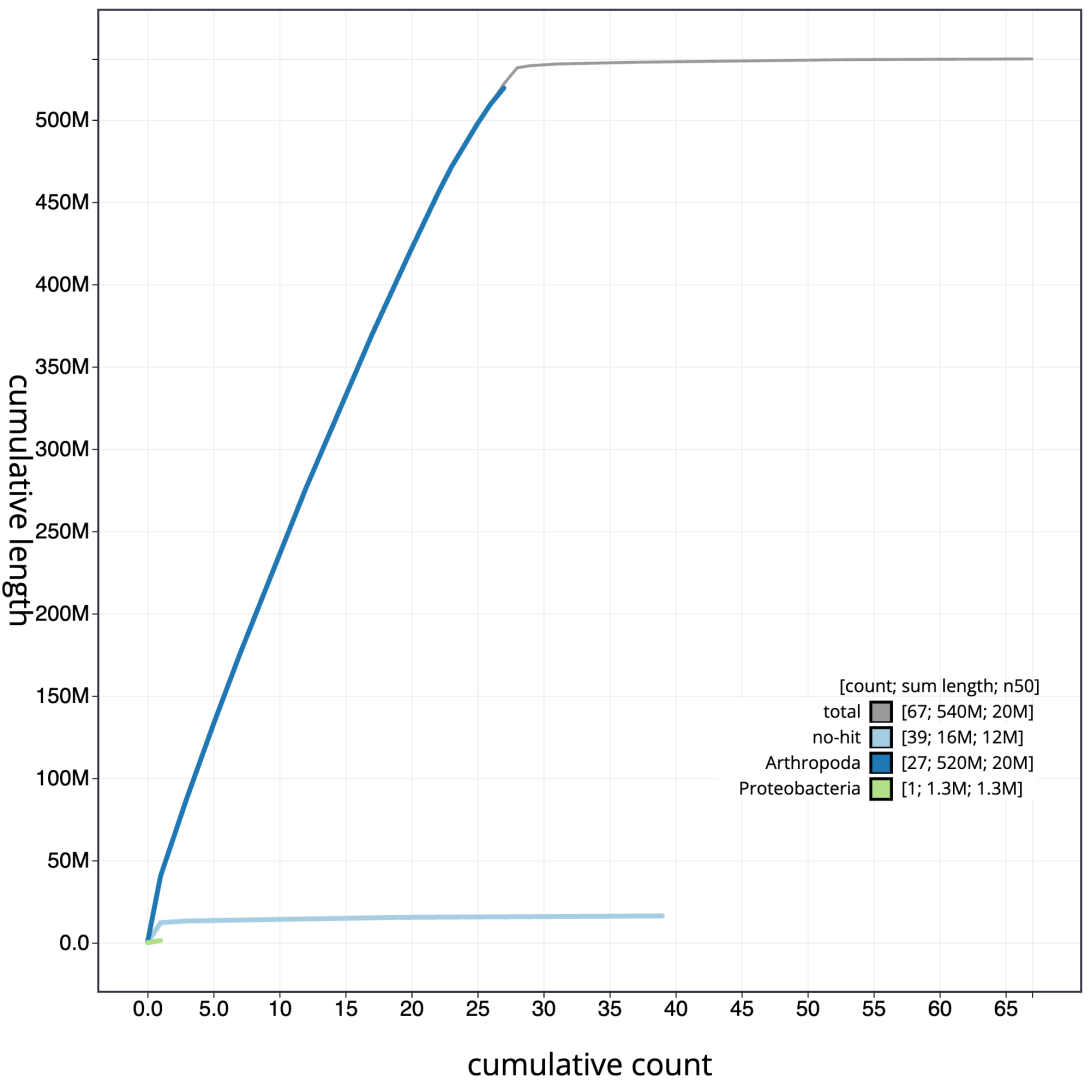
Genome assembly of *Thymelicus acteon*, ilThyActe1.1: BlobToolKit cumulative sequence plot. The grey line shows cumulative length for all sequences. Coloured lines show cumulative lengths of sequences assigned to each phylum using the buscogenes taxrule. An interactive version of this figure is available at https://blobtoolkit.genomehubs.org/view/ilThyActe1_1/dataset/ilThyActe1_1/cumulative.

**Figure 5.**
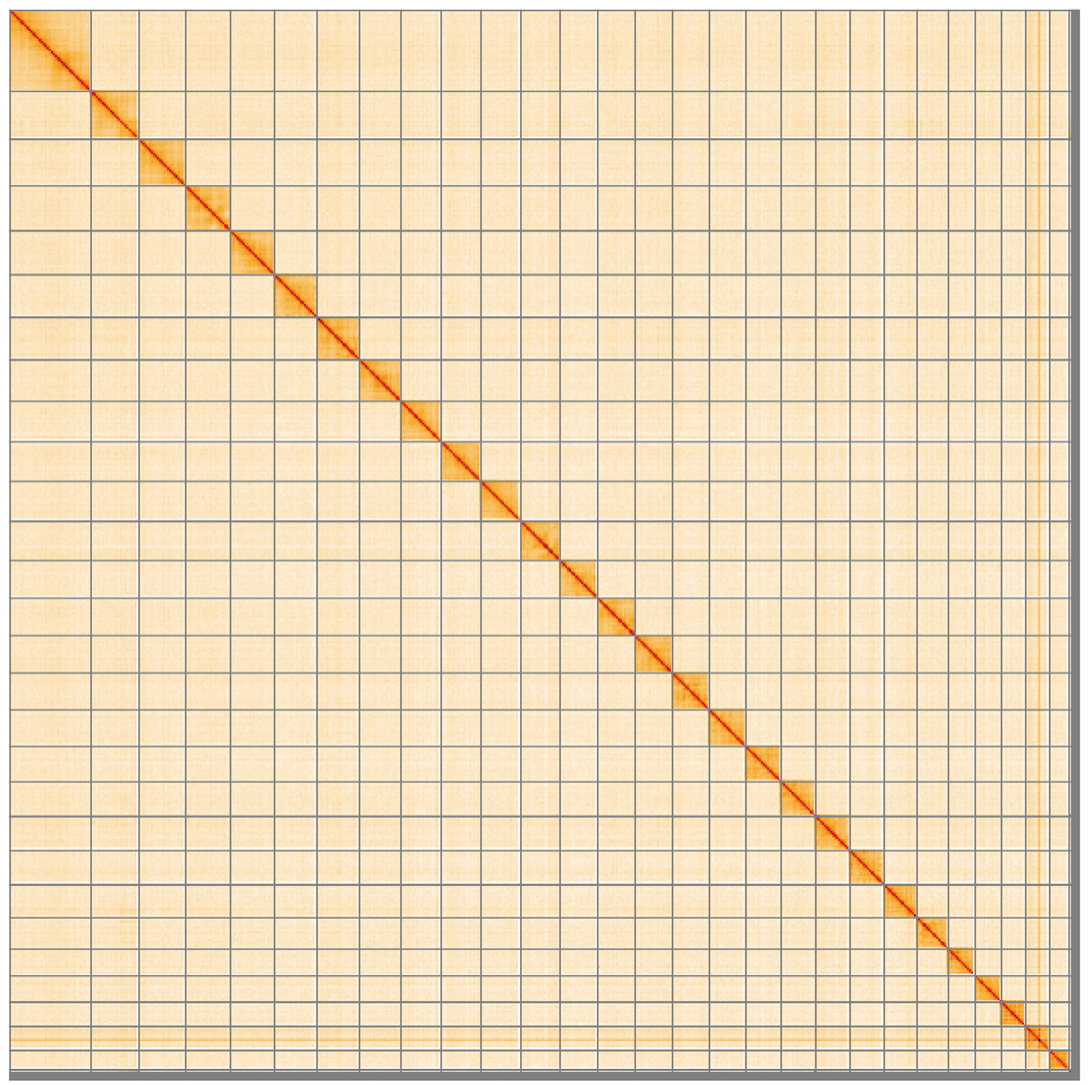
Genome assembly of *Thymelicus acteon*, ilThyActe1.1: Hi-C contact map of the ilThyActe1.1 assembly, visualised using HiGlass. Chromosomes are shown in order of size from left to right and top to bottom. An interactive version of this figure may be viewed at https://genome-note-higlass.tol.sanger.ac.uk/l/?d=DwelZModSOCAr3bDC4wEig.

The final assembly has a total length of 537.0 Mb in 66 sequence scaffolds with a scaffold N50 of 19.7 Mb ( Table 1). The snail plot in Figure 2 provides a summary of the assembly statistics, while the distribution of assembly scaffolds on GC proportion and coverage is shown in Figure 3. The cumulative assembly plot in Figure 4 shows curves for subsets of scaffolds assigned to different phyla. Most (98.98%) of the assembly sequence was assigned to 28 chromosomal-level scaffolds, representing 27 autosomes and the Z sex chromosome. Chromosome-scale scaffolds confirmed by the Hi-C data are named in order of size ( Figure 5; Table 2). While not fully phased, the assembly deposited is of one haplotype. Contigs corresponding to the second haplotype have also been deposited. The mitochondrial genome was also assembled and can be found as a contig within the multifasta file of the genome submission.

**Table 1.** Genome data for *Thymelicus acteon*, ilThyActe1.1.

Project accession data		
Assembly identifier	ilThyActe1.1	
Species	*Thymelicus acteon*	
Specimen	ilThyActe1	
NCBI taxonomy ID	876078	
BioProject	PRJEB59958	
BioSample ID	SAMEA110069525	
Isolate information	ilThyActe1, male: head (PacBio sequencing) ilThyActe2, male: head (Hi-C sequencing)	
Assembly metrics *		*Benchmark*
Consensus quality (QV)	65.0	*≥ 50*
*k*-mer completeness	100.0%	*≥ 95%*
BUSCO **	C:98.4%[S:98.0%,D:0.5%],F:0.4%,M:1.2%,n:5,286	*C ≥ 95%*
Percentage of assembly mapped to chromosomes	98.98%	*≥ 95%*
Sex chromosomes	Z	*localised homologous pairs*
Organelles	Mitochondrial genome: 17.08 kb	*complete single alleles*
Raw data accessions		
PacificBiosciences Sequel IIe	ERR10906098	
Hi-C Illumina	ERR10908636	
Genome assembly		
Assembly accession	GCA_951805285.1	
*Accession of alternate haplotype*	GCA_951805295.1	
Span (Mb)	537.0	
Number of contigs	120	
Contig N50 length (Mb)	10.7	
Number of scaffolds	66	
Scaffold N50 length (Mb)	19.7	
Longest scaffold (Mb)	40.67	
Genome annotation		
Number of protein-coding genes	12,813	
Number of non-coding genes	2,647	
Number of gene transcripts	25,933	

* Assembly metric benchmarks are adapted from column VGP-2020 of “Table 1: Proposed standards and metrics for defining genome assembly quality” from Rhie *et al.* (2021). ** BUSCO scores based on the lepidoptera_odb10 BUSCO set using version 5.3.2. C = complete [S = single copy, D = duplicated], F = fragmented, M = missing, n = number of orthologues in comparison. A full set of BUSCO scores is available at https://blobtoolkit.genomehubs.org/view/ilThyActe1_1/dataset/ilThyActe1_1/busco.

**Table 2.** Chromosomal pseudomolecules in the genome assembly of *Thymelicus acteon*, ilThyActe1.

INSDC accession	Chromosome	Length (Mb)	GC%
OX638213.1	1	23.4	36.5
OX638214.1	2	24.07	37.0
OX638215.1	3	22.12	36.5
OX638216.1	4	22.48	36.5
OX638217.1	5	21.31	36.5
OX638218.1	6	21.33	36.0
OX638219.1	7	20.81	36.0
OX638220.1	8	20.0	36.0
OX638221.1	9	20.19	36.0
OX638222.1	10	20.0	36.5
OX638223.1	11	19.68	36.0
OX638224.1	12	18.87	36.0
OX638225.1	13	18.82	36.0
OX638226.1	14	18.75	36.0
OX638227.1	15	18.52	36.5
OX638228.1	16	18.31	36.0
OX638229.1	17	17.7	36.0
OX638230.1	18	17.2	36.0
OX638231.1	19	17.38	36.5
OX638232.1	20	17.12	36.5
OX638233.1	21	16.51	36.5
OX638234.1	22	15.74	36.5
OX638235.1	23	13.45	36.0
OX638236.1	24	13.2	36.5
OX638237.1	25	12.18	36.5
OX638238.1	26	12.01	37.0
OX638239.1	27	9.73	36.5
OX638212.1	Z	40.67	35.5
OX638240.1	MT	0.02	16.5

The estimated Quality Value (QV) of the final assembly is 65.0 with *k*-mer completeness of 100.0%, and the assembly has a BUSCO v5.3.2 completeness of 98.4% (single = 98.0%, duplicated = 0.5%), using the lepidoptera_odb10 reference set ( *n* = 5,286).

Metadata for specimens, barcode results, spectra estimates, sequencing runs, contaminants and pre-curation assembly statistics are given at https://links.tol.sanger.ac.uk/species/876078.

## Genome annotation report

The *Thymelicus acteon* genome assembly (GCA_951805285.1) was annotated at the European Bioinformatics Institute (EBI) on Ensembl Rapid Release. The resulting annotation includes 25,933 transcribed mRNAs from 12,813 protein-coding and 2,647 non-coding genes ( Table 1; https://rapid.ensembl.org/Thymelicus_acteon_GCA_951805285.1/Info/Index).

## Methods

### Sample acquisition and nucleic acid extraction

Two male *Thymelicus acteon* specimens were collected from El Serrat, Catalunya, Spain (latitude 41.69, longitude 2.25) on 2017-05-28 using a sweep net. The specimen used for genome sequencing had ID SAN00002425 (ToLID ilThyActe1), while the specimen used for Hi-C sequencing had ID SAN00002426 (ToLID ilThyActe2). Both specimens were collected by Konrad Lohse (University of Edinburgh) and Alex Hayward (University of Exeter) and identified by Roger Vila (Universitat Pompeu Fabra). Samples were snap frozen at –80 °C in a dry shipper.

The workflow for high molecular weight (HMW) DNA extraction at the Wellcome Sanger Institute (WSI) Tree of Life Core Laboratory includes a sequence of core procedures: sample preparation; sample homogenisation, DNA extraction, fragmentation, and clean-up. In sample preparation, the ilThyActe1 sample was weighed and dissected on dry ice ( Jay *et al.*, 2023). Tissue from the head was homogenised using a PowerMasher II tissue disruptor ( Denton *et al.*, 2023a).

HMW DNA was extracted in the WSI Scientific Operations core using the Automated MagAttract v2 protocol ( Oatley *et al.*, 2023). The DNA was sheared into an average fragment size of 12–20 kb in a Megaruptor 3 system with speed setting 31 ( Bates *et al.*, 2023). Sheared DNA was purified by solid-phase reversible immobilisation ( Strickland *et al.*, 2023): in brief, the method employs a 1.8X ratio of AMPure PB beads to sample to eliminate shorter fragments and concentrate the DNA. The concentration of the sheared and purified DNA was assessed using a Nanodrop spectrophotometer and Qubit Fluorometer and Qubit dsDNA High Sensitivity Assay kit. Fragment size distribution was evaluated by running the sample on the FemtoPulse system.

Protocols developed by the WSI Tree of Life laboratory are publicly available on protocols.io ( Denton *et al.*, 2023b).

### Sequencing

Pacific Biosciences HiFi circular consensus DNA sequencing libraries were constructed according to the manufacturers’ instructions. DNA sequencing was performed by the Scientific Operations core at the WSI on a Pacific Biosciences Sequel IIe instrument. Hi-C data were also generated from head tissue of ilThyActe2 using the Arima2 kit and sequenced on the Illumina NovaSeq 6000 instrument.

### Genome assembly, curation and evaluation

Assembly was carried out with Hifiasm ( Cheng *et al.*, 2021) and haplotypic duplication was identified and removed with purge_dups ( Guan *et al.*, 2020). The assembly was then scaffolded with Hi-C data ( Rao *et al.*, 2014) using YaHS ( Zhou *et al.*, 2023). The assembly was checked for contamination and corrected as described previously ( Howe *et al.*, 2021). Manual curation was performed using HiGlass ( Kerpedjiev *et al.*, 2018) and PretextView ( Harry, 2022). The mitochondrial genome was assembled using MitoHiFi ( Uliano-Silva *et al.*, 2023), which runs MitoFinder ( Allio *et al.*, 2020) or MITOS ( Bernt *et al.*, 2013) and uses these annotations to select the final mitochondrial contig and to ensure the general quality of the sequence.

A Hi-C map for the final assembly was produced using bwa-mem2 ( Vasimuddin *et al.*, 2019) in the Cooler file format ( Abdennur & Mirny, 2020). To assess the assembly metrics, the *k*-mer completeness and QV consensus quality values were calculated in Merqury ( Rhie *et al.*, 2020). This work was done using Nextflow ( Di Tommaso *et al.*, 2017) DSL2 pipelines “sanger-tol/readmapping” ( Surana *et al.*, 2023a) and “sanger-tol/genomenote” ( Surana *et al.*, 2023b). The genome was analysed within the BlobToolKit environment ( Challis *et al.*, 2020) and BUSCO scores ( Manni *et al.*, 2021; Simão *et al.*, 2015) were calculated.

Table 3 contains a list of relevant software tool versions and sources.

**Table 3.** Software tools: versions and sources.

Software tool	Version	Source
BlobToolKit	4.2.1	https://github.com/blobtoolkit/blobtoolkit
BUSCO	5.3.2	https://gitlab.com/ezlab/busco
Hifiasm	0.16.1-r375	https://github.com/chhylp123/hifiasm
HiGlass	1.11.6	https://github.com/higlass/higlass
Merqury	MerquryFK	https://github.com/thegenemyers/MERQURY.FK
MitoHiFi	2	https://github.com/marcelauliano/MitoHiFi
PretextView	0.2	https://github.com/wtsi-hpag/PretextView
purge_dups	1.2.3	https://github.com/dfguan/purge_dups
sanger-tol/genomenote	v1.0	https://github.com/sanger-tol/genomenote
sanger-tol/readmapping	1.1.0	https://github.com/sanger-tol/readmapping/tree/1.1.0
YaHS	1.1a.2	https://github.com/c-zhou/yahs

### Genome annotation

The Ensembl Genebuild annotation system ( Aken *et al.*, 2016) was used to generate annotation for the *Thymelicus acteon* assembly (GCA_951805285.1) in Ensembl Rapid Release at the EBI. Annotation was created primarily through alignment of transcriptomic data to the genome, with gap filling via protein-to-genome alignments of a select set of proteins from UniProt ( UniProt Consortium, 2019).

### Wellcome Sanger Institute – Legal and Governance

The materials that have contributed to this genome note have been supplied by a Darwin Tree of Life Partner. The submission of materials by a Darwin Tree of Life Partner is subject to the **‘Darwin Tree of Life Project Sampling Code of Practice’**, which can be found in full on the Darwin Tree of Life website here. By agreeing with and signing up to the Sampling Code of Practice, the Darwin Tree of Life Partner agrees they will meet the legal and ethical requirements and standards set out within this document in respect of all samples acquired for, and supplied to, the Darwin Tree of Life Project.

Further, the Wellcome Sanger Institute employs a process whereby due diligence is carried out proportionate to the nature of the materials themselves, and the circumstances under which they have been/are to be collected and provided for use. The purpose of this is to address and mitigate any potential legal and/or ethical implications of receipt and use of the materials as part of the research project, and to ensure that in doing so we align with best practice wherever possible. The overarching areas of consideration are:

•      Ethical review of provenance and sourcing of the material

•      Legality of collection, transfer and use (national and international)

Each transfer of samples is further undertaken according to a Research Collaboration Agreement or Material Transfer Agreement entered into by the Darwin Tree of Life Partner, Genome Research Limited (operating as the Wellcome Sanger Institute), and in some circumstances other Darwin Tree of Life collaborators.

## Data Availability

European Nucleotide Archive: *Thymelicus acteon* (Lulworth skipper). Accession number PRJEB59958; https://identifiers.org/ena.embl/PRJEB59958 ( Wellcome Sanger Institute, 2023). The genome sequence is released openly for reuse. The *Thymelicus acteon* genome sequencing initiative is part of the Darwin Tree of Life (DToL) project. All raw sequence data and the assembly have been deposited in INSDC databases. Raw data and assembly accession identifiers are reported in Table 1.

## Author information

Members of the Wellcome Sanger Institute Tree of Life Management, Samples and Laboratory team are listed here: https://doi.org/10.5281/zenodo.10066175.

Members of Wellcome Sanger Institute Scientific Operations: Sequencing Operations are listed here: https://doi.org/10.5281/zenodo.10043364.

Members of the Wellcome Sanger Institute Tree of Life Core Informatics team are listed here: https://doi.org/10.5281/zenodo.10066637.

Members of the Tree of Life Core Informatics collective are listed here: https://doi.org/10.5281/zenodo.5013541.

Members of the Darwin Tree of Life Consortium are listed here: https://doi.org/10.5281/zenodo.4783558.
