# Supplementary material for: The genome sequence of the Lulworth Skipper, Thymelicus acteon (Rottemburg, 1775)
Source: Wellcome Open Res. 2024 May 15;9:266. [Version 1] doi: 10.12688/wellcomeopenres.21627.1 (PMC11384194; doi:10.12688/wellcomeopenres.21627.1)
Supplement: Peer review history

# Reviewer report: Reviewer response for version 1

**DOI:** 10.21956/wellcomeopenres.23899.r94328

**Published:** 2024-09-11

**Authors:** Xiaoling Fan

**Affiliations:** 1 South China Agricultural University, Guangzhou, Guangdong, China

The type of species of the genus *Thymelicus* , *T. acteo* n, includes several subspecies. The study specifically focuses on the genomic sequencing of the nominate subspecies of *T. acteon* , based on the information available from the collected specimen.

 The research only involves genomic sequencin, assembly, and annotation. These techniques are well-established in modern biological research. The genomic data obtained from the nominate subspecies will provide crucial insights into the taxonomic status of the species and its various subspecies within the genus. This study merits indexing if it meets the journal’s criteria.

Are sufficient details of methods and materials provided to allow replication by others?

Yes

Is the rationale for creating the dataset(s) clearly described?

Yes

Are the datasets clearly presented in a useable and accessible format?

Yes

Are the protocols appropriate and is the work technically sound?

Yes

Reviewer Expertise:

Systematics of Hesperiidae

I confirm that I have read this submission and believe that I have an appropriate level of expertise to confirm that it is of an acceptable scientific standard.

# Reviewer report: Reviewer response for version 1

**DOI:** 10.21956/wellcomeopenres.23899.r94329

**Published:** 2024-09-09

**Authors:** Li-Wei Wu

**Affiliations:** 1 Tunghai University, Taichung City, Taiwan

This manuscript provides a genome information about the Lulworth skipper, *Thymelicus acteon*. The length of this male genome is 537 Mb in length, and total 28 chromosome-level pseudomolecules are assembled, including Z sex chromosome. The assembly quality, with an N50 of 19.7 Mb and a BUSCO completeness of 98.4%, indicates a highly complete genomic dataset.

 The use of Pacific Biosciences HiFi long reads and Hi-C technology for assembly correction greatly enhances the utility of this genome for further studies in conservation genetics, evolution and phylogeography, making it an essential reference for future genomic analyses.

 The analysis presented in this manuscript provides excellent genomic information. However, there are aspects of the background that could be enhanced to increase application of this manuscript and help readers, who interest this genome, understand the biology of the species more comprehensively. Two suggestions as follow:

1. The second paragraph of the background section states, "but in the UK it has increased in abundance since 2014, possibly as a result of climate change." This claim should be supported by additional citations to clarify why the authors consider climate change a possible factor affecting the abundance of this skipper.
2. Regarding the mitochondrial genome, which has been assembled to a length of 17-Kb, it would be beneficial to provide the GenBank accession number. This would offer convenient public access to the data, allowing others to utilize this information directly (not just only the SRA datasets). Overall, these enhancements could solidify its usefulness and accessibility.

Are sufficient details of methods and materials provided to allow replication by others?

Yes

Is the rationale for creating the dataset(s) clearly described?

Yes

Are the datasets clearly presented in a useable and accessible format?

Yes

Are the protocols appropriate and is the work technically sound?

Yes

Reviewer Expertise:

conservation genetics, phylogeny, historical biogeography

I confirm that I have read this submission and believe that I have an appropriate level of expertise to confirm that it is of an acceptable scientific standard.

# Reviewer report: Reviewer response for version 1

**DOI:** 10.21956/wellcomeopenres.23899.r92909

**Published:** 2024-08-23

**Authors:** Elena Pazhenkova

**Affiliations:** 1 University of Ljubljana, Ljubljana, Slovenia

The article presents a comprehensive genome assembly of the Lulworth Skipper, *Thymelicus acteon* (Lepidoptera, Hesperiidae). The authors constructed a high-quality assembly using data from PacBio HiFi long reads and Hi-C data. The resulting genome sequence spans 537 Mb, with an assembly of 28 chromosomal pseudomolecules, including the Z sex chromosome. Additionally, the mitochondrial genome, spanning 17 Kb, has been assembled.

 Gene annotation using Ensembl identified a total of 12813 protein-coding genes, providing valuable insights into the genetic makeup of the Lulworth Skipper. The study includes detailed taxonomic information, habitat description, and phenotypic variation across its range, enhancing the context of the research. The article also highlights the ecological significance and conservation status of the species.

 Overall, this article provides a valuable resource for researchers interested in the biology of the Lulworth Skipper. The genome assembly presented in this study serves as a solid foundation for further investigations into the species' adaptation, population genomics, and conservation strategies. The comprehensive data provided makes this article an important contribution to the field of biodiversity research and genomics.

Are sufficient details of methods and materials provided to allow replication by others?

Yes

Is the rationale for creating the dataset(s) clearly described?

Yes

Are the datasets clearly presented in a useable and accessible format?

Yes

Are the protocols appropriate and is the work technically sound?

Yes

Reviewer Expertise:

Entomology, conservation, evolutionary biology

I confirm that I have read this submission and believe that I have an appropriate level of expertise to confirm that it is of an acceptable scientific standard.
